# The College of Nursing (Limited by Guarantee)

**Published:** 1921-03-26

**Authors:** 


THE
### COLLEGE OF NURSING
(Limited by Guarantee.)

### What the Local Centres are Doing.

### CORNWALL CENTRE, TRURO.

(Hon. Secretary, Miss E. M. Jordan, Convalescent Homes, Perranporth.)

Wednesday, April 6, 3.30 p.m.—Annual Meeting at the Royal Cornwall Infirmary, Truro (by kind permission). The election of officers for the ensuing year will take place. It is expected that Miss Sheriff MacGregor and Miss Billing, Matron of Torbay Hospital, will speak. Miss Billing is the Centre's candidate for the forthcoming Council election, and all members are asked to make a special effort to be present to meet her.

### NORTHUMBERLAND AND DURHAM CENTRE, NEWCASTLE-ON-TYNE.

(Secretary, Miss M. Toyne, 17 Windsor Terrace, Jesmond.)

At the meeting of the members of this Centre, held at the Nurses' Club, 17 Windsor Terrace, on Friday, March 18, Miss M. Herbert (retired hospital matron), and Hon. Secretary of this Centre, was nominated as a candidate for the forthcoming Council election.

The Annual Meeting is to be held on Saturday, April 16, at 3 p.m., when the election of new members in place of the retiring members of the Executive Committee will take place.

Members of this Centre are reminded that their annual subscription of 5s. is due on April 1, and are asked to send it to the Secretary, 17 Windsor Terrace, on or as soon as possible after that date.

### EDINBURGH CENTRE.

(Hon. Secretary, Miss Cathcart, The Elms, Whitehouse Loan.)

Nurses' Club Social Evenings.

The Social Committee of the Edinburgh Nurses' Club has arranged for the first gathering to take the form of a whist-drive, which will be held in the Club, 8 Drumsheugh Gardens, on Thursday, March 31, at 7.30 p.m. To cover the cost of refreshments and expenses the charge, for this first evening, has been fixed at 1s. 6d. per head, but it is hoped that in the future it may be less. This will naturally depend on the number of members who support the movement. It is proposed for the future to hold these social evenings on the last Friday of every month unless any other plan is substituted during the summer months.

Mrs. Bowie has kindly consented to act as Secretary to the Committee. Members intending to be present on March 31 are asked to intimate this not later than March 28 to Mrs. Bowie, at 8 Drumsheugh Gardens, Edinburgh. Tickets will not be issued, but the charge for expenses will be taken on arrival.

Annual Subscriptions.

Club subscriptions are now due, and should be sent to the Lady Superintendent, Edinburgh Nurses' Club, 8 Drumsheugh Gardens, if possible on or before March 31, 1921.

### GLASGOW CENTRE.

(Hon. Secretary, Miss E. Moseley, Western District Hospital, Oakbank.)

Saturday, March 26, 3 p.m.—In the Glasgow Nurses' Club (Miss Roy Reid), 10 Claremont Terrace, W., lecture on "Nursing Treatment of Throat, Nose, and Ear Cases," by J. Harper, Esq., M.A., M.B., Ch.B., Surgeon to the Royal Infirmary. Members are invited to bring nurse friends.

Saturday, April 2, 3 p.m.—An American Tea will be held at 10 Claremont Terrace.

